# A Computational Model of Deep Brain Stimulation for Parkinson’s Disease Tremor and Bradykinesia

**DOI:** 10.3390/brainsci14060620

**Published:** 2024-06-20

**Authors:** Sandeep Sathyanandan Nair, Srinivasa Chakravarthy

**Affiliations:** 1 Department of Biotechnology, Bhupat and Mehta Jyoti School of Biosciences, Chennai 600036, India; sandeepsathyanandan@gmail.com; 2 Department of Medical Science and Technology, Indian Institute of Technology Madras, Sardar Patel Road, Adyar, Chennai 600036, India

**Keywords:** deep brain stimulation (DBS), sub thalamic nucleus, basal ganglia, dopamine, Parkinson’s disease, motor symptoms, tremor, rigidity, bradykinesia, theta, beta

## Abstract

Parkinson’s disease (PD) is a progressive neurological disorder that is typically characterized by a range of motor dysfunctions, and its impact extends beyond physical abnormalities into emotional well-being and cognitive symptoms. The loss of dopaminergic neurons in the substantia nigra pars compacta (SNc) leads to an array of dysfunctions in the functioning of the basal ganglia (BG) circuitry that manifests into PD. While active research is being carried out to find the root cause of SNc cell death, various therapeutic techniques are used to manage the symptoms of PD. The most common approach in managing the symptoms is replenishing the lost dopamine in the form of taking dopaminergic medications such as levodopa, despite its long-term complications. Another commonly used intervention for PD is deep brain stimulation (DBS). DBS is most commonly used when levodopa medication efficacy is reduced, and, in combination with levodopa medication, it helps reduce the required dosage of medication, prolonging the therapeutic effect. DBS is also a first choice option when motor complications such as dyskinesia emerge as a side effect of medication. Several studies have also reported that though DBS is found to be effective in suppressing severe motor symptoms such as tremors and rigidity, it has an adverse effect on cognitive capabilities. Henceforth, it is important to understand the exact mechanism of DBS in alleviating motor symptoms. A computational model of DBS stimulation for motor symptoms will offer great insights into understanding the mechanisms underlying DBS, and, along this line, in our current study, we modeled a cortico-basal ganglia circuitry of arm reaching, where we simulated healthy control (HC) and PD symptoms as well as the DBS effect on PD tremor and bradykinesia. Our modeling results reveal that PD tremors are more correlated with the theta band, while bradykinesia is more correlated with the beta band of the frequency spectrum of the local field potential (LFP) of the subthalamic nucleus (STN) neurons. With a DBS current of 220 pA, 130 Hz, and a 100 microsecond pulse-width, we could found the maximum therapeutic effect for the pathological dynamics simulated using our model using a set of parameter values. However, the exact DBS characteristics vary from patient to patient, and this can be further studied by exploring the model parameter space. This model can be extended to study different DBS targets and accommodate cognitive dynamics in the future to study the impact of DBS on cognitive symptoms and thereby optimize the parameters to produce optimal performance effects across modalities. Combining DBS with rehabilitation is another frontier where DBS can reduce symptoms such as tremors and rigidity, enabling patients to participate in their therapy. With DBS providing instant relief to patients, a combination of DBS and rehabilitation can enhance neural plasticity. One of the key motivations behind combining DBS with rehabilitation is to expect comparable results in motor performance even with milder DBS currents.

## 1. Introduction

Parkinson’s disease (PD) is a severe neurodegenerative disease that affects a large percentage of the older human population and, at present, is second only to Alzheimer’s in terms of the number of people it affects [1,2,3]. With the progression of the disease, PD manifests with some of the cardinal symptoms such as tremors, rigidity, bradykinesia, and even loss of balance [3,4,5,6,7,8]. The onset and progression of the disease have a close link to neuronal loss in the substantia nigra pars compacta (SNc) [9,10,11] that unsettles the dopaminergic pathways in the basal ganglia (BG). When contemplating symptom management strategies, the predominant approach often involves replenishing dopamine loss by administering dopaminergic medications such as levodopa (LDOPA) [12,13]. Another commonly used technique is stimulating the relevant brain region using an external current with appropriate characteristics, known as deep brain stimulation (DBS) [14,15,16]. Since prolonged usage of dopaminergic medications leads to motor complications like dyskinesias and the wearing-off effect, the number of people undergoing DBS surgery has increased significantly. Research suggests that DBS is beneficial in cases where the effectiveness of medication diminishes or severe side effects occur and in reducing medication dosage [17]. Furthermore, when combined with LDOPA, it provides a highly effective therapeutic approach [18,19].

DBS entails an electrode surgically implanted into the skull, delivering an electrical current with precise parameters into the subcortical region [20]. While dopaminergic medications like levodopa focus on rectifying dopamine deficiency within the BG pathways [21], DBS targets specific functional regions of the pallido-subthalamic circuitry and serves as a catalyst for exploration within the cortico-basal ganglia circuitry, which is essential for learning. This exploration is mainly facilitated through the modulation of the pallido-subthalamic circuitry, which constitutes the indirect pathway of the basal ganglia. From a functional connectivity standpoint, cortical inputs reach the BG-striatum’s input port, which then channels information to the output nucleus, GPi, through two pathways—one directly and another via the pallido-subthalamic circuitry [22]. By applying DBS to the STN, an integral component of this circuitry, the intricate dynamics of information flow and modulation within the basal ganglia thalamocortical network are further elucidated.

Numerous studies have observed increased beta-band oscillations and beta power in the STN region in patients with PD [23]. Experimental research has revealed that the interplay between cortical and basal ganglia (BG) structures, including the pallidum and subthalamic nucleus (STN), can induce beta rhythm oscillations throughout the cortico-basal ganglia system. These oscillations are commonly seen during PD symptoms [24,25,26,27]. The low dopamine levels lead to synchronous firings of both STN and GPe neurons [28,29]. Theoretical studies have shown the relationship between neuronal synchrony and collateral strengths in STN and GPe neurons [30,31]. DBS application mitigates synchronous neuronal communication and partially restores the normal functioning of the indirect pathway circuitry, contributing to improved behavioral outcomes.

To delve into the dynamics of pallido-subthalamic circuitry, initial studies focused on analyzing the various firing characteristics by modulating the synaptic strength and connectivity patterns [32]. Later, this single-compartment biophysical model was expanded [32] by incorporating the globus pallidus interna (GPi) and thalamus [33]. The relationship between PD tremor and the oscillations in pallido-subthalamic circuitry was discussed in various experimental studies [24,34,35,36,37]. Also, the relationship between the STN and cortical oscillations was explored [38].

While the aforementioned models provide insights into neural activity and dynamics, it is crucial to comprehend how these effects translate into behavior, as the ultimate aim of DBS is the well-being of individuals with PD. Modeling behavior allows for a comprehensive assessment of the diverse impacts of PD and the potency of interventions like DBS. PD affects various behaviors, including motor functions such as tremors, rigidity, impaired coordination, and movement precision, as well as cognitive functions like decision making and executive control. The impact of DBS on cognitive symptoms has been explored using a computational model of spiking neurons [39,40]; it was found that stimulation of STN worsened the decision-making performance in tasks such as the Iowa Gambling task.

Regarding motor tasks, modeling arm-reaching tasks has particular significance. Reaching tasks are pivotal in daily activities and are intricately linked with motor symptoms experienced by patients with PD. By simulating these tasks, researchers can gain insights into the specific motor deficits present in PD and evaluate the effectiveness of interventions like DBS in restoring motor function and improving overall motor performance. Considering this literature, the objectives of the proposed study were (i) to understand the origins of PD tremors in the cortico-basal ganglia circuit, (ii) to understand the effect of DBS on PD tremors and (iii) to optimize the DBS parameters to minimize tremor.

This outline of the paper is as follows: Section 2 discusses the materials and methods, which describe the cortico-basal ganglia model and its key components. We then describe the mechanism used to simulate the PD condition, followed by the DBS intervention. Section 3 highlights some of our essential results from the model, and then Section 4 provides a discussion. 

## 2. Materials and Methods

**Figure 1:**
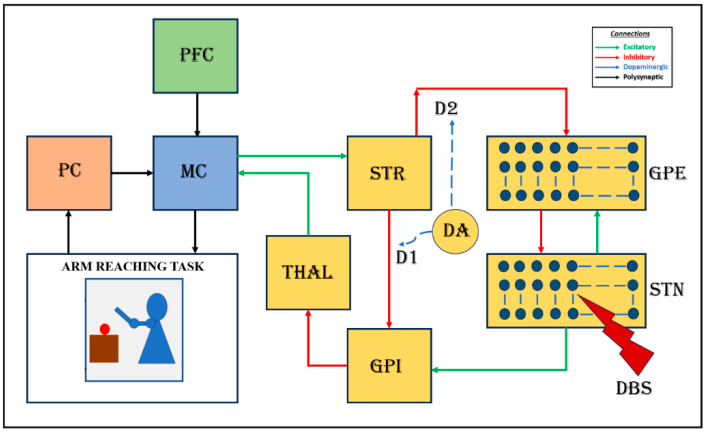
Block diagram of the proposed cortico-basal ganglia model. The model consists of a 2-link arm model, the proprioceptive cortex (PC), the prefrontal cortex (PFC), the motor cortex (MC), and the basal ganglia (BG). Here, the input nucleus striatum, the output nucleus globus pallidus internus (GPi), the globus pallidus externus (GPe), the subthalamic nucleus (STN), and the thalamus (THAL) constitute the BG. MC integrates the inputs received from the prefrontal cortex (PFC) and the proprioceptive cortex (PC) along with the feedback signal from BG and sends the signal to the arm via the spinal motor neurons.

We used a cortico-BG (CBG) model that controls a two-linked arm model to simulate reaching movements, as shown in Figure 1. In this section, we first introduce the CBG model and the DBS intervention. The earliest efforts in modeling coordinated reaching movements started with control-system-based loops [41,42,43,44,45]. While this research did not focus on the underlying neural mechanisms, parallel studies were conducted on the neural substrates and neural mechanisms involved in reaching [46,47,48]. Soon, reinforcement-learning-based models were in use [49]. In our study, an arm model [49] was integrated into a basal ganglia thalamocortical model consisting of the oscillatory model of the STN–GPe network [50] to simulate motor movements at the behavioral level [51,52]. To accommodate the DBS effect on performance, we replaced the rate-coded model of pallido-subthalamic circuitry with a spiking neuron model and studied DBS effects. We describe the arm model in the Supplementary Materials Section.

### 2.1. Cortico-Basal Ganglia Model

The CBG model used in our study comprised an outer sensory-motor loop, which interacted with the BG circuitry. The sensory-motor loop comprised an arm model, the proprioceptive cortex (PC), the motor cortex (MC), the prefrontal cortex (PFC), and the BG. The kinematic arm model performed reaching movements based on the activations it received, and the PC estimated the current arm position and sent the feedback to the MC, which integrated this signal along with the goal information from the PFC and the error-corrected signal from the BG, which conducted its internal processing before sending back the corrected signal to the motor cortex. The MC then sent the following motor command to the arm via the spinal motor neurons, and this process continued until the arm reached the target or the time out was reached. More details about the sensory-motor loop and the arm model are discussed in the Supplementary Materials. Please note that we use the term PC because, in the current model, we only considered the proprioceptive input and no other sensory inputs. Otherwise, the anatomically correct usage would be the primary somatosensory cortex. The MC acts as the intersection layer between the outer sensory motor loop and the BG circuitry. Communication between the MC and the BG is crucial to exploring and understanding PD dynamics.

The BG consists of the striatum (STR), which is the input nucleus; globus pallidus internus (GPi), which is the output nucleus; and the globus pallidus externus (GPe) and STN, which constitute the indirect pathway. The subthalamic nucleus (STN) and globus pallidus interna (GPi), which constitute the indirect pathway, were modeled using the spiking neuron model (Izhikevich). In contrast, the rest of the BG nuclei were modeled using the rate-coded model.

#### 2.1.1. MC–BG Interaction

Effective interaction between the BG and cortex is crucial in motor acquisition and performance. This interplay facilitates learning, leading to decision-making scenarios where competing signals—one facilitating and the other inhibiting—are evaluated. This evaluation forms the basis of selecting the best action and is facilitated by the GPi mechanism [53,54], where inputs via two parallel pathways, the D1 and D2, combine. The flow of information through the D1 and the D2 pathways is modulated by the dopamine signal from the SNc [22]. The depletion of dopamine signals due to neuronal death results in motor impairments, which are then managed using dopaminergic medication or DBS.

The MC receives inputs from the PFC, the PC, and the BG, and the total input received at the MC, $I^{MC}$, is as given in Equation (1). The dynamics of PC and PFC are given in the Supplementary Materials Section.

(1) $$I^{MC}=A^{PFC}\cdot G^{PFC}+A^{PC}\cdot G^{PC}+A^{BG}\cdot G^{BG}$$

The dynamics of the MC neurons are defined by the continuous attractor neural network (CANN), and the MC output ($G^{MC}$) is as given in Equation (2):

(2) $$G^{MC}\left(t\right)=\frac{{\left(g^{MC}\right)}^{2}}{1+\left(\frac{2\pi }{{\left(N^{MC}\right)}^{2}}\right)b^{MC}\sum {\left(g^{MC}\right)}^{2}}$$

where $N^{MC}$ defines the network size of MC, $b^{MC}$ is a constant, and $g^{MC}$ represents the intrinsic state of the nodes in MC and is as given in Equation (3).

(3) $$\tau_{MC}\frac{dg^{MC}}{dt}=-g^{MC}+W_{MC}^{C}\;⨂\;G^{MC}+I^{MC}$$

where the weight kernel $W_{MC}^{C}$ represents the lateral connectivity among the MC neurons, and ⨂ is the convolutional operation.

The MC input is presented to the striatum (STR), which routes the signal to the GPi directly as well as through the STN–GPe network. The signal projecting from the D1 medium spiny neurons (MSNs) of the striatum to GPi is $y^{D1},\;$as given in Equation (4), whereas the signal projecting from the D2 MSNs of the striatum to GPe is given as $y^{D2}$ in Equation (5). The $D1_{\lambda }$ and $D2_{\lambda }$ in Equations (6) and (7) represent the sigmoidal activation of the *D*1 and *D*2 striatal neurons.

(4) $$y^{D1}=D2_{\lambda }W^{MC\to D1}\Delta G_{MC}$$

(5) $$y^{D2}=D2_{\lambda }W^{MC\to D2}\Delta G_{MC}$$

(6) $$D1_{\lambda }=\frac{1}{1+\exp\left(-k_{D1}\left(\delta_{val}-\phi_{D1}\right)\right)}$$

(7) $$D2_{\lambda }=\frac{1}{1+\exp\left(-k_{D2}\left(\delta_{val}-\phi_{D2}\right)\right)}$$

where the weights $W^{MC\to D1}\;$and $W^{MC\to D2}$ represent the weights between the MC and STR, $\Delta G_{MC}$ is the input received by the STR from MC, $\delta_{val}$ is the value difference that modulates the BG pathways, and $k_{D1}$ and $k_{D2}$ are the sigmoidal gains, where $k_{D1}=-k_{D2}$ and $\phi_{D1}$ and $\phi_{D2}$ are the thresholds used in the sigmoidal function for $D1_{\lambda }$ and $D2_{\lambda }$, respectively.

The quantity $\delta_{val}$ used here is termed the value difference, and it is computed as given in Equation (8) below.

(8) $$\delta_{val}=V^{arm}(t)-V^{arm}(t-1)$$

$V^{arm}\left(t\right)$ in Equation (8) represents the value of the current position of the arm at time ‘*t*’ and is obtained as the probabilistic gradient ascent over the value function [22,55] performed by the BG, as given in Equation (9) below. $X^{arm}$ and $X^{targ}$ are the current arm position obtained from the PC and the target goal position obtained from the PFC, respectively. The value function, $V^{arm}\left(t\right)$, is obtained as given in Equation (9).

(9) $$V^{arm}\left(t\right)=exp\left(\frac{-{\left\|X^{targ}-X^{arm}\right\|}^{2}}{\sigma_{V}^{2}}\right)$$

where the spatial distance within which the value function demonstrates sensitivity for that particular target is given by $\sigma_{V}$.

#### 2.1.2. The STN–GPe Subsystem

The D2 MSNs of the STR project to the GPe, and the current received at the GPe neurons from the STR is as given in Equation (10).

(10) $$I_{ij}^{StrD2\to GPe}=A^{GPe}\cdot y^{D2}$$

Here, $A^{GPe}$ represents the weight between the STR and GPe. The total incoming current at the GPe is as described in Equation (11).

(11) $$I_{ij}^{GPe}\left(t\right)=I_{ij}^{GABAlat}\left(t\right)+I_{ij}^{NMDA\to GPe}\left(t\right)+I_{ij}^{AMPA\to GPe}\left(t\right)+{\;I}_{ij}^{StrD2\to GPe}\left(t\right)+I^{GPe}(t)$$

The GPe neurons also receive input from their own neighboring neurons, which is given in Equation (12), and the corresponding neurons in the STN, as given in Equations (13) and (14).

(12) $$I_{ij}^{GABAlat}\left(t\right)=\sum_{p,q=1}^{n}W_{ij,pq}^{GPe\to GPe}\left(t\right)*h_{ij}^{GABA\to GPe}\left(t\right)\cdot (E_{GABA}-V_{GPe})$$

(13) $$I_{ij}^{NMDA\to GPe}\left(t\right)=W_{SG}.\;h_{ij}^{NMDA\to GPe}\left(t\right)\cdot (E_{NMDA}-V_{GPe})$$

(14) $$I_{ij}^{AMPA\to GPe}\left(t\right)=W_{SG}.\;h_{ij}^{AMPA\to GPe}\left(t\right)\cdot (E_{AMPA}-V_{GPe})$$

In the above equations, $V_{GPe}$ represents the voltage across the membrane of the GPe neurons, as described in Equations (15)–(17). The STN–GPe network consists of 2D arrays of spiking neurons modeled using Izhikevich equations. When the membrane voltage reaches $V^{Peak}$, the variables are reset, as shown below.

(15) $$\frac{dV_{ij}^{GPe}}{dt}=\left(0.04\right){\left(V_{ij}^{GPe}\right)}^{2}+5V_{ij}^{GPe}-U_{ij}^{x}+140+I_{ij}^{GPe\;}+y^{D2}$$

(16) $$\frac{dU_{ij}^{GPe}}{dt}=a^{gpe}(b^{gpe}V_{ij}^{GPe}-U_{ij}^{GPe})$$

(17) $$if\;V_{ij}^{GPe}>V^{Peak},\;then\;V_{ij}^{GPe}=c^{GPe}\;;\;U_{ij}^{GPe}=U_{ij}^{GPe}+d^{GPe}$$

Also, $h_{ij}^{GABA\to GPe},h_{ij}^{NMDA\to GPe},$ and $h_{ij}^{AMPA\to GPe}$ represent the gating variables, as shown in Equations (18)–(20).

(18) $$\tau_{Recep}*dh_{ij}^{NMDA\to GPe}\left(t\right)=-h_{ij}^{NMDA\to GPe}\left(t\right)\cdot S_{ij}^{GPe}(t)$$

(19) $$\tau_{Recep}*dh_{ij}^{AMPA\to GPe}\left(t\right)=-h_{ij}^{AMPA\to GPe}\left(t\right)\cdot S_{ij}^{GPe}(t)$$

(20) $$\tau_{Recep}*dh_{ij}^{GABA\to GPe}\left(t\right)=-h_{ij}^{GABA\to GPe}\left(t\right)\cdot S_{ij}^{GPe}(t)$$

$I^{GS}\;$is the current from the GPe neurons to the corresponding STN neurons, as shown in Equation (21). Here, $W_{GS}\;$denotes the weights between the STN and GPe neurons, $h_{ij}^{GABA\to STN}$ regulates the gating between the GABA and STN, $V^{STN}$ is the voltage across the membrane for the STN neurons, and $E_{GABA}$ is the voltage across the membrane at rest for the GPe neurons.

(21) $$I_{ij}^{GS}\left(t\right)=W_{GS}.\;h_{ij}^{GABA\to STN}\left(t\right)\cdot (E_{GABA}-V^{STN})$$

Along with $I^{GS}$, the lateral currents from the neighboring STN neurons constitute the total current received at each STN neuron, which is governed by Equations (22)–(24).

(22) $$I_{ij}^{STN}\left(t\right)=I_{ij}^{GS}\left(t\right)+I_{ij}^{NMDA\to STN}\left(t\right)+I_{ij}^{AMPA\to STN}\left(t\right)$$

(23) $$I_{ij}^{AMPAlat}\left(t\right)=\sum_{p,q=1}^{n}W_{ij,pq}^{STN\to STN}\left(t\right)*h_{ij}^{AMPA\to STN}\left(t\right)\cdot (E_{AMPA}-E_{STN})$$

(24) $$I_{ij}^{NMDAlat}\left(t\right)=B_{ij}\left(v\right)*\sum_{p,q=1}^{n}W_{ij,pq}^{STN\to STN}\left(t\right)*h_{ij}^{NMDA\to STN}\left(t\right)\cdot (E_{NMDA}-E_{STN})$$

In the above equations, the terms $h_{ij}^{AMPA\to STN}$, $h_{ij}^{NMDA\to STN}$, and $h_{ij}^{GABA\to STN}$ represent the gating variables, and their dynamics are as shown in Equations (25)–(27).

(25) $$\tau_{Recep}*dh_{ij}^{NMDA\to STN}\left(t\right)=-h_{ij}^{NMDA\to GPe}\left(t\right)\cdot S_{ij}^{STN}(t)$$

(26) $$\tau_{Recep}*dh_{ij}^{AMPA\to STN}\left(t\right)=-h_{ij}^{AMPA\to GPe}\left(t\right)\cdot S_{ij}^{STN}(t)$$

(27) $$\tau_{Recep}*dh_{ij}^{GABA\to STN}\left(t\right)=-h_{ij}^{GABA\to STN}\left(t\right)\cdot S_{ij}^{STN}(t)$$

The membrane potentials of the STN neurons are described in Equations (28)–(30). The terms $h_{ij}^{AMPA\to STN}$, $h_{ij}^{NMDA\to STN}$, and $h_{ij}^{GABA\to STN}$ represent gating variables, and their dynamics are as shown in the following equations:

(28) $$\frac{dV_{ij}^{STN}}{dt}=\left(0.04\right){\left(V_{ij}^{STN}\right)}^{2}+5V_{ij}^{STN}-U_{ij}^{x}+140+I_{ij}^{STN\;}$$

(29) $$\frac{dU_{ij}^{STN}}{dt}=a^{STN}(b^{STN}V_{ij}^{STN}-U_{ij}^{STN})\;$$

(30) $$if\;V_{ij}^{STN}>V^{Peak},\;then\;V_{ij}^{STN}=c^{STN}\;;\;U_{ij}^{STN}=U_{ij}^{STN}+d^{STN}$$

The local field potential (LFP) of the STN is calculated as shown in Equation (31).

(31) $${LFP}^{STN}={\left(\frac{1}{R_{ij}^{MON}}\right)}^{2}\cdot (I_{ij}^{GS}\left(t\right)+I_{ij}^{NMDA\to STN}\left(t\right)+I_{ij}^{AMPA\to STN}\left(t\right))$$

The term $R_{ij}^{MON}$ in the above equation is the distance between the ${\left(i,\;j\right)}^{th}$ neuron and the recording point.

#### 2.1.3. Simulating the PD Condition

To replicate the symptoms of PD, the DA value was decreased from $DA_{HC\;}$ to $DA_{low}$. In other words, we prevented the $\delta_{val}$ from rising beyond $DA_{low}$, as given in Equation (32).

If PD = 1 then,

(32) $$\delta_{val}\left(t\right)=min(\delta_{val}\left(t\right),DA_{low})$$

The DA value also influences the lateral connections of the STN and GPe nucleus and the interconnectivity ($W_{SG}\;and\;W_{GS})$ between the corresponding neurons of the STN and GPe, as shown in Equations (33)–(37).

(33) $$W_{ij,pq}^{m\to m}=W_{m}^{max\;}*\exp\left(-\frac{{d_{ij,pq}}^{2}}{R_{m}}\right);\;{d_{ij,pq}}^{2}={\left(i-p\right)}^{2}+{\left(j-q\right)}^{2}\;$$

where m in the above equation represents the STN/GPe. $W_{m}^{max\;}$ is the maximum connectivity strength among the neurons, $R_{m}$ defines the radius of the neighborhood, *d* is the distance between two neurons in the subpopulation, and (*i*, *j*, *p*, *q*) represent the indices of the neurons.

(34) $$R_{STN}=10-9.89\frac{\left(DA-0.1\right)}{0.8};R_{GPe}=0.22+19.78\frac{\left(DA-0.1\right)}{0.8}$$

(35) $$W_{SG}=7.92+1.18\frac{\left(DA-0.1\right)}{0.8};W_{GS}=29.7+170.5\frac{\left(DA-0.1\right)}{0.8}\;$$

(36) $$I^{GPe}=3+3\frac{\left(DA-0.1\right)}{0.8}$$

(37) $$W_{ij,pq}^{STN\to STN}=W_{STN}^{max\;}\exp\left(-\frac{{d_{ij,pq}}^{2}}{R_{m}}\right);\;{d_{ij,pq}}^{2}={\left(i-p\right)}^{2}+{\left(j-q\right)}^{2}\;$$

For simulating the tremor and rigidity conditions, we modulated the connectivity strength between then STN and GPi using the gain factor $A^{D2}$, mentioned in Equation (38) and Equation (39). For tremor, a relatively higher value (2) of $A^{D2}$ was chosen, whereas for rigidity, a lower value (<0.4) of $A^{D2}$ was chosen.

#### 2.1.4. The STN-to-GPi Connection

Inputs from then STN to GPi are taken after converting the spike data of STN neurons into rate codes. The mean rate of firing of the STN neurons is calculated as shown in Equation (38).

(38) $$y^{STN}\left(t\right)=\frac{\sum_{T_{init}}^{t}{\sum_{i=1}^{NI}\sum_{j=1}^{NJ}1.SPK}_{ij}^{STN}}{{(N}_{STN}\cdot N_{STN})}\;$$

where $y^{STN}\;isthe\;$average firing rate of the STN neurons for a simulation time of 1 s, ${SPK}_{ij}^{STN}$ is the spike data of neuron at location (i, j) in the network, N is the total number of neurons (*NI* × *NJ*), and *T* = simulation time (1 s). $T_{init}=t-WS$ if t is greater than or equal to the WS; otherwise, $T_{init}=t$. Here, WS is the temporal window size.

#### 2.1.5. The BG-to-MC Connection

The input through the direct projections from the D1 MSNs of the STR $\left(Y^{D1}\right)$ and the output of the STN $\left(y^{STN}\right)$ are combined at GPi $\left(y^{GPi}\right),$ as shown in Equation (39), before forwarding to the thalamus.

(39) $$Y^{GPI}=A^{D1}y^{D1}+A^{D2}y^{STN}$$

The dynamics of the thalamic neurons are modeled as a CANN, and the thalamic output is as given by Equation (40),

(40) $$G^{thal}\left(t\right)=\frac{{\left(g^{thal}\right)}^{2}}{1+\left(\frac{2\pi }{{\left(N^{thal}\right)}^{2}}\right)b^{thal}\sum {\left(g^{thal}\right)}^{2}}$$

where $N_{thal}$ defines the thalamic network size, $b_{thal}$ is a constant, $A^{D1}\;\mathrm{and}\;A^{D2}$ are the respective gains associated with the two pathways, and $g_{thal}$ represents the intrinsic state of thalamic neurons as given by Equation (41).

(41) $$\tau_{thal}\frac{dg_{thal}}{dt}=-g^{thal}+W_{thal}^{C}\;⨂\;G^{thal}+I^{BG}$$

(42) $$I^{BG}=Y^{GPi}\;$$

(43) $$G^{BG}=G^{thal}\;$$

where the weight kernel, $W_{thal}^{C}$, represents the lateral connectivity strength among the thalamic neurons; $I^{BG}$ is the input from the GPi to thalamus coming from the BG; $Y^{GPi}$ and $G^{thal}\;$are outputs of the GPi and thalamus, respectively; and $G^{BG}$ is the thalamic output to the MC.

### 2.2. Parameter Selection

The list of parameters used in the model and their corresponding values, along with their description, is given in the Supplementary Materials Section. Also, the learning mechanisms are described in detail in the Supplementary Materials Section. The newly added parameter sets for behavioral manifestation, STN–GPe dynamics, and DBS parameters are provided in Tables S2–S4 of the Supplementary Materials Section, respectively. The timing parameter (each time step of the MC loop) was set to 0.0125 s in order to tune the model to match the performance of the HC.

### 2.3. DBS Effect

There are various targets used for DBS, and, among them, the GPi and ventralis intermedialis (Vim) of the thalamus are commonly used in addition to the STN [20,56,57]. However, STN DBS is most commonly used for PD [16], considering its better therapeutic effects.

The pulsatile current of the appropriate parameters (amplitude, frequency, and pulse duration) mimicking the clinically delivered DBS [58] effect was simulated in our model. The current is applied to the centermost neuron (position in the 2D lattice ($i_{m}$, $j_{m}$), and the spread of the current to the neighboring neurons is modulated by a Gaussian distribution [59,60] with variance ($\sigma_{DBS}$), as shown in Equation (44).

(44) $$I_{DBS}^{ij}\left(t\right)=exp\left(\frac{-{\left((i-i_{m}\right)}^{2}+{\left(j-j_{m}\right)}^{2})}{\sigma_{DBS\;}^{2}}\right)$$

where $I_{DBS}^{ij}\left(t\right)\;$is the effective *DBS* current received by the neuron at location (*i*, *j*).

### 2.4. Informing the Model Using Experimental Data

We used experimental reaching performance data from [61] to inform our model. These data included kinematic parameters such as movement time, peak velocity, and time to peak velocity recorded during a reaching task performed by six healthy controls (HC) and six patients with PD diagnosed with stage three Parkinson’s disease on the H&Y [62] scale. Participants were asked to reach and grasp an object placed a certain distance away. Similar trends were observed in [63], where participants (16 with HC and 14 with PD) performed reaching movements toward visually presented targets. Another study on 11 participants by [64] involved visually guided reaching tasks that included upper limb and eye movements. The upper limb reaching performance (PD reaching time) in patients with PD observed in [64] was similar to that in [61]. The comparison between the model performance and the experimental results of the kinematic parameters is shown in the Results Section (Section 3.5). We followed the below steps to validate our model”

(i). Data Source: The experimental reaching performance data from [61] was used for behavior modeling.
(ii). Experimental Task: The participants were asked to reach and grasp a ball that was placed a certain distance away from them as quickly as possible.
(iii). Parameter Tuning for HC and PD Groups: Model parameters were adjusted to replicate the reaching performance of healthy controls (HC).
(iv). Firing Rate Calibration: While tuning parameters in step (ii), we ensured that the firing rates of the subthalamic nucleus (STN) and globus pallidus externus (GPe) matched the experimental data [39,65,66] observed in both HC and PD conditions.
(v). Frequency Spectrum Analysis: The STN local field potentials (LFPs) and their frequency spectrum dynamics were analyzed and compared with experimental data [67,68,69,70].
(vi). The network dynamics were then correlated with the symptoms.

## 3. Results

Here, we showcase the model’s performance by simulating the healthy (HC) and PD conditions and observing their effects on the arm-reaching task. In order to simulate the HC and PD conditions, we modulated the dopamine-dependent control parameters in our cortico-basal ganglia model neuron model, especially the amount of current flowing from the D1 and D2 striatal neurons, and the parameters in the STN–GPe subsystem as detailed in the section below.

### 3.1. Parameters Controlling the Firing Patterns and Synchrony in STN–GPe

The neuronal firings and synchrony of the STN and GPe populations of neurons were tuned using various parameters, such as the lateral connection spread of the GPe neurons ($R_{GPe})$, the lateral connection spread of the STN neurons ($R_{STN})$, the spread of laterals in STN and GPe neurons, the interconnections between STN and GPe ($W_{SG}\;and\;W_{GS})$, the dopamine availability, the striatum to GPe current ($I^{GPe}),$ and the STN current. Dopamine signal (DA) modulates the lateral connectivity of both the STN and GPe populations, the interconnections between the two neuronal populations, and the input current to GPe, as shown in Equations (34)–(37).

### 3.2. Kinematic Performance of Arm Reaching

**Figure 2:**
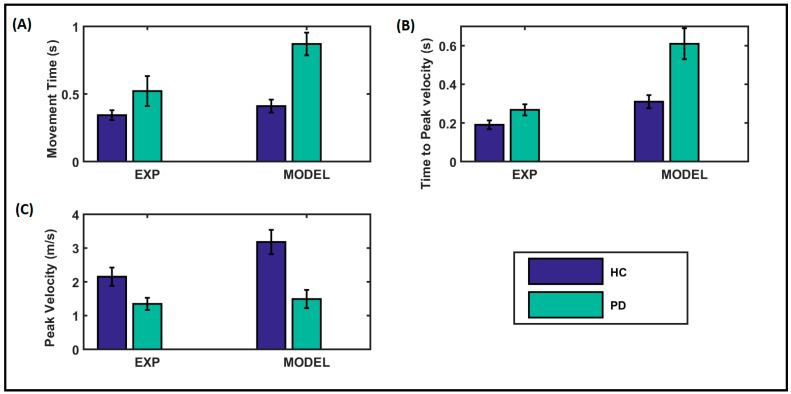
Comparison of performance of the proposed model with experimental data adapted from [61]. (**A**) Movement time, (**B**) time-to-peak velocity, (**C**) peak velocity; sec, second; m/s, meter per second. The dark blue bar represents the healthy control (HC) group, and the green bar represents the PD condition.

The parameters for reaching performance in our model for healthy control (HC) participants and patients with PD were tuned to match the experimental performance data taken from [61], where the parameters of movement time, peak velocity, and time to peak velocity were recorded during a reaching task. The comparison between the model performance and the experimental results is shown in Figure 2. below. The recordings were taken over an average of five trials. The experimental and the model results showed a similar trend, where the movement time was comparatively higher for the PD condition. HC’s peak velocity was comparatively higher in both experimental and model results. In contrast, the time to peak velocity was higher under PD conditions, as reflected in both the experimental and model results.

### 3.3. Neuronal Firings and Synchrony during Healthy and PD Conditions

**Figure 3:**
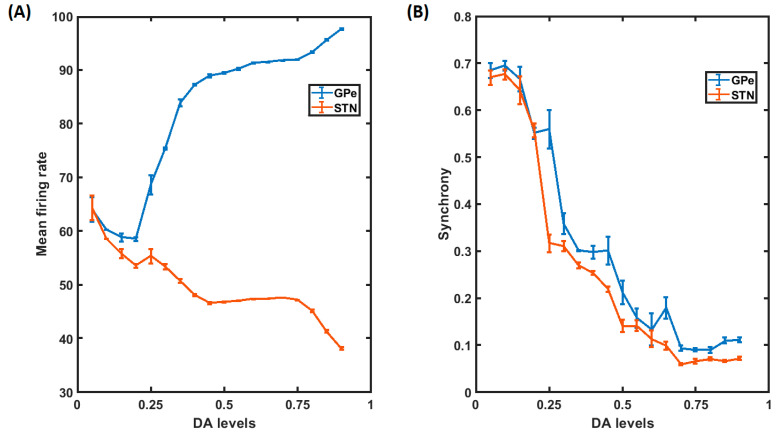
Firing rates and synchrony. (**A**) The firing rates of STN and GPe neurons for various values of DA levels are shown. The blue line represents the GPe, and the orange line represents the STN neurons. (**B**) Synchrony within STN and GPe nuclei. Again, blue and orange lines represent the GPe and STN neurons, respectively. Synchrony keeps decreasing with increasing DA levels. The mean and variance values for the above plots were calculated over five epochs.

The variations in firing rate and synchrony to dopamine levels are shown in Figure 3. The parameters were tuned to match the STN and GPe firing rates to the experimental values [39,65,66]. As the dopamine level increased from 0.1 to 0.9, the firing rates of the STN neurons decreased. In contrast, the firing rates of the GPe neurons increased (Figure 3A). Also, the synchrony among the neurons of both STN and GPe decreased with increasing dopamine levels (Figure 3A). The recordings were taken over an average of five trials.

**Figure 4:**
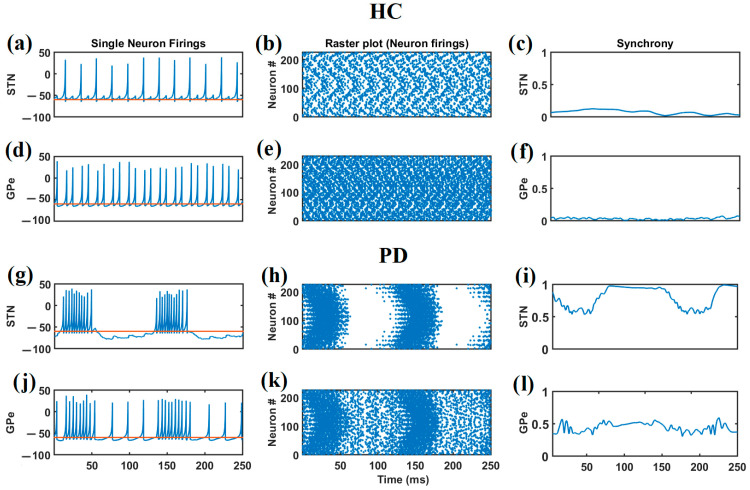
The firing of single STN and GPe neurons in the HC group is shown in (**a**,**d**). Under HC conditions, both the STN and GPe neurons exhibit regular firings. The firing of single STN and GPe neurons under PD conditions is shown in (**g**,**j**). (**b**,**e**) The raster plot of the STN, and (**h**,**k**) the GPe neurons in PD. (**c**,**f**) the synchrony of STN and GPe neurons under healthy conditions, and (**i**,**l**) the synchrony of STN and GPe neurons under PD condition. The orange lines in (**a**,**d**,**g**,**j**) indicate the reference line corresponding to the theoretical resting membrane potential (-60 mV) and the blue line represents the spike data.

We selected two DA values, 0.1 and 0.9, to simulate the PD and HC conditions. The neuronal firings and synchrony of the STN and GPe populations of neurons under both these conditions are given in Figure 4. Figure 4a–f present the dynamics under the healthy condition, whereas Figure 4g–l present the dynamics of the PD condition. Under the healthy control (HC) condition, both the STN and GPE neurons exhibit regular spiking, as shown in Figure 4a,d, and the activities of the neuronal subpopulation in both the STN and GPe exhibit asynchronous firings, as shown in Figure 4b,e. Figure 4c,f show the synchrony among the STN and GPe neuronal population as a function of time. During the PD condition, the dopaminergic neurons in the SNc die, and, in our model, we simulated the same by reducing the dopamine level from 0.9 to 0.1. The reduction in dopamine level influences the STN–GPe circuitry via the lateral connections of the STN and GPe and the interconnections between the STN and GPe. Figure 4g,j show that due to this influence, the STN and GPe fire in burst mode, and there is increased synchrony among both the STN and GPe neurons, as shown in Figure 4h,k. Figure 4c,f,i,l present the synchrony among the neurons of the respective neuronal populations.

### 3.4. Arm Reaching Performance under Healthy and PD Conditions

**Figure 5:**
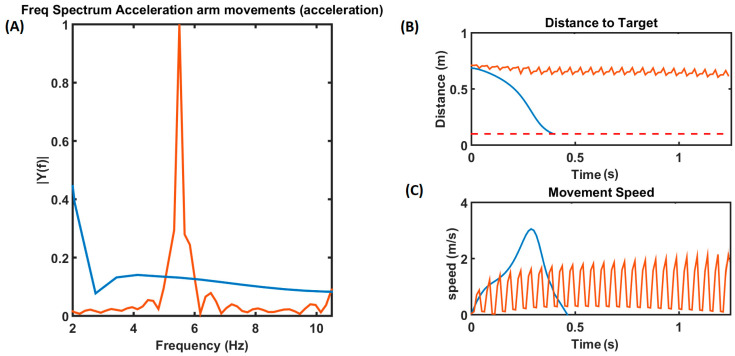
(**A**) The frequency spectrum of the acceleration of the arm movements. The blue line represents the healthy control (HC) condition, and the orange line represents the PD tremor condition in all (**A**–**C**). (**B**) This plot shows the distance to the target as the time progresses. (**C**) The velocity of the arm movement, where the curve follows a bell curve under HC conditions and keeps oscillating under PD tremor conditions.

Reaching movements were simulated using the model described in Figure 1 with the DA level set to 0.9. The blue line in Figure 5 represents the reaching performance of the HC group. It can be seen that during the control condition (HC), the arm consistently reached the target, with the velocity of the arm forming a bell curve in Figure 5C over time, where the speed increases until it reaches a peak and then slowly reduces as the arm approaches the target. The distance to target slowly reduces, resembling a waterfall curve, as it reaches the target (Figure 5B). Acceleration of the arm during the reaching task was as shown in Figure 5A, where we do not see any significant peaks in the tremor frequency (4–10 Hz) band in the spectrum of the arm acceleration, while we do see a significant peak around 7 Hz in case of the PD tremor condition, represented by the orange line in Figure 5A. Under the PD tremor condition, we can also see that the arm never reaches the target, and it keeps fluctuating as shown in the orange curve in Figure 5B. Also, the velocity of the arm keeps increasing and decreasing while the tremor is experienced, as shown in the orange curve in Figure 5C.

**Figure 6:**
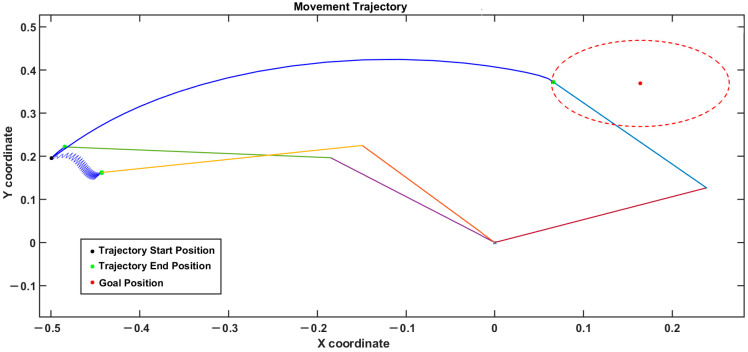
The trajectory of the arm movements is given. The blue line (for the arm) represents the healthy control (HC) condition, the yellow line (for the arm) represents the tremor condition, and the green line (for the arm) represents the rigidity condition. In the case of HCs, the reaching is successful, whereas in the case of tremor and rigidity, it is not.

The trajectory of the arm is shown in Figure 6. For the controls (HC), the arm consistently reached the target, as indicated by the blue arm, whereas during PD tremor conditions, the arm kept fluctuating, as shown by the yellow arm. During a rigidity case, the arm hardly moved from the starting position, as shown by the green arm. The red dotted circle around the target position is the region where the arm is considered to have reached the target within its area.

**Figure 7:**
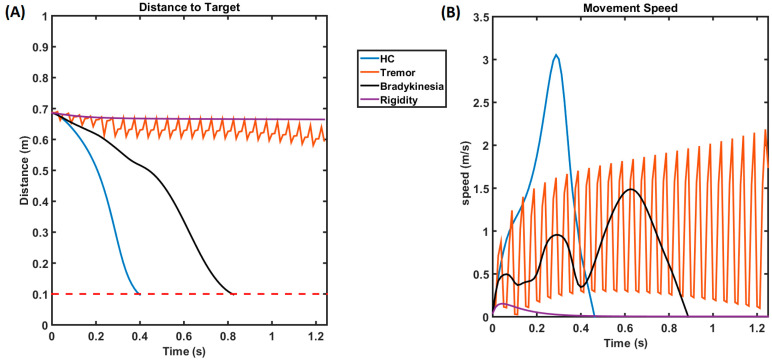
(**A**) This plot shows the distance to the target as the time progresses. (**B**) This plot shows the velocity of the arm movement, where the curve follows a bell curve for the HCs, has multiple lesser-magnitude peaks under the bradykinesia condition, and keeps oscillating under the PD tremor condition. The arm hardly moves, and the velocity curve quickly decreases down under rigidity conditions. The blue line represents the healthy controls (HCs), the purple line represents the rigidity condition, the orange line represents the tremor condition, and the black line represents the bradykinesia condition.

For the HCs, the distance to the target of the arm steadily decreased and reached the target (~0.4 s), as shown in the blue line in Figure 7A, while under tremor conditions, we can also see that the arm never reached the target, and it kept fluctuating, as shown in the orange curve. In the case of rigidity, the arm hardly moved, and the distance to the target was a steady, flat line, as shown by the purple curve. In the case of bradykinesia, the arm took a comparatively longer time to reach the target (~0.8375 s). Figure 7B shows the velocity of the arm while performing the reaching, and, as seen, the blue line representing HC resembles a bell curve, where the velocity increases until it peaks and then gradually decreases. Also, the velocity of the arm kept increasing and decreasing under the tremor condition (orange line) and hardly raised under the rigidity condition (purple line). In the case of bradykinesia (black line), the velocity curve exhibits multiple peaks of comparatively lesser amplitude, which is a characteristic of bradykinesia.

**Figure 8:**
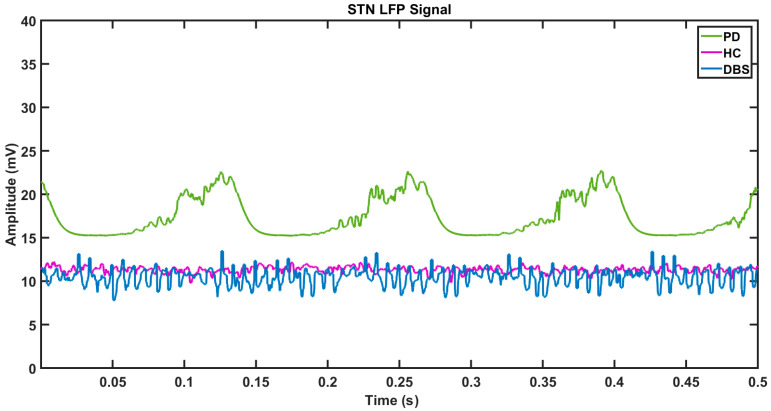
The potential of the STN neuronal population in the local field is shown. The violet curve indicates the HC condition, the green line indicates the PD condition, and the blue line represents the DBS-treated condition.

**Figure 9:**
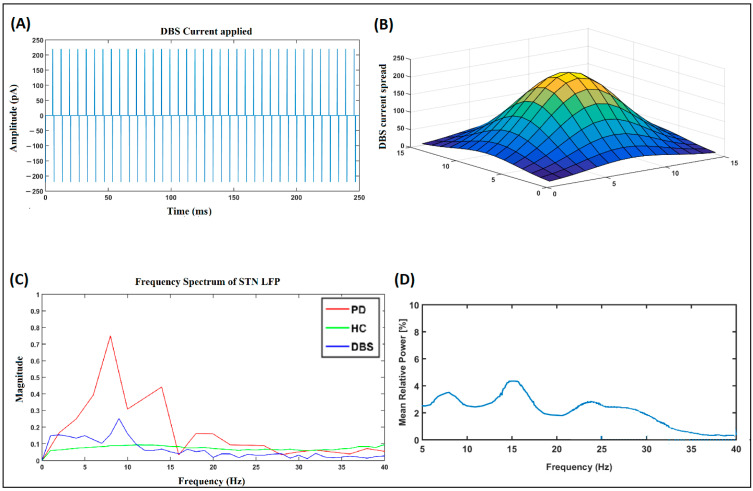
(**A**) DBS is currently applied to the center of most neurons in the STN population. (**B**) The spread of the current in nearby neurons. (**C**) The FFT of the local field potential of the STN population. (**D**) The mean relative power of the LFP of the STN was redrawn as recorded in the experimental studies (Kuhn et al., 2008) [68].

During the tremor condition, the neuronal population of the STN subsystem fires in a synchronous manner, and, hence, the local field potential (LFP) of the STN is highly periodic and higher in amplitude (green line), as shown in Figure 8, whereas the amplitude of the LFP signal in the HC and DBS-treated conditions, represented by violet and blue lines, is comparatively much smaller. Looking closely at the dynamics of the STN–GPe, the frequency spectrum of the LFP of the STN neuron population reveals that there was a significantly higher power observed in the beta frequency band (13–35 Hz), as shown in Figure 9C. During the tremor condition, the beta peaks were also accompanied by another peak at the theta band (4–11 Hz). This is in line with the observations in an experimental study [68].

### 3.5. Simulating Deep Brain Stimulation (DBS) Effect

As significant beta peaks in the LFP of the STN are a signature of PD, attempts have been made to suppress this beta peak. The DBS facilitates the suppression of beta and theta peaks by injecting a high-frequency current of appropriate amplitude and pulse duration. The DBS current used in our simulation is shown in Figure 9A. A biphasic pulsatile current of 130 Hz, 220 pA, and 100 microsecond pulse duration was applied to the centermost neuron of the STN subpopulation, and the effect of the DBS current on the neighboring neurons was modeled as a Gaussian spread, as shown in Figure 9B. In our study, we checked the impact of STN DBS on reaching performance under the tremor condition. The current was applied to the centermost neuron. We used biphasic, single-contact stimulation.

### 3.6. Effect of DBS on PD Symptoms (Tremor and Bradykinesia)

**Figure 10:**
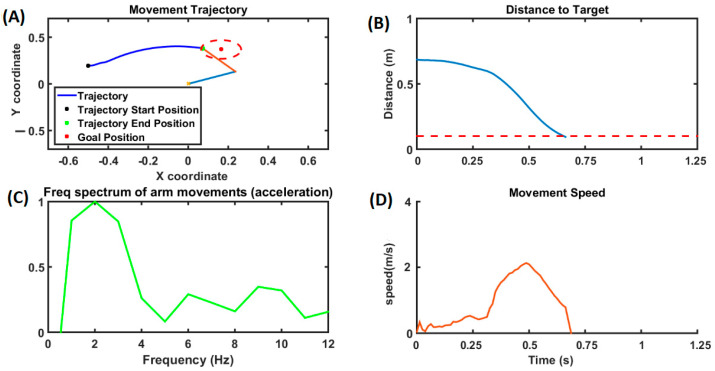
The movement trajectory of the arm movements is given in (**A**), where the blue line represents the trajectory and the red dot represents the target position. The distance to target over time is shown in (**B**), where as the frequency spectrum of the acceleration of arm movements is shown in (**C**) and the velocity of arm movements is shown in (**D**).

The DBS stimulation restored the reaching performance of the arm movement, as shown in Figure 10. We can also observe that compared to the HC performance in Figure 5 and Figure 6, the performance still shows a vast improvement, and the arm was able to reach the target in slightly more time (0.8 s) than in the HCs (0.4 s). Figure 8A shows the movement trajectory for a DBS-treated condition, and it can be seen that the arm reached the target after a period of time. Figure 10B shows the distance to the target as a function of time, and we can see that the distance to the target kept decreasing with time. Figure 10C shows the frequency spectrum of the acceleration of the arm movements during the reaching task, and we can see that the power in the band region between 4 and 10 Hz was significantly reduced. Figure 10D shows the velocity curve during reaching movement, and it can be seen that it took a while to attain the peak velocity, which was followed by a gradual reduction until the arm reached the target.

**Figure 11:**
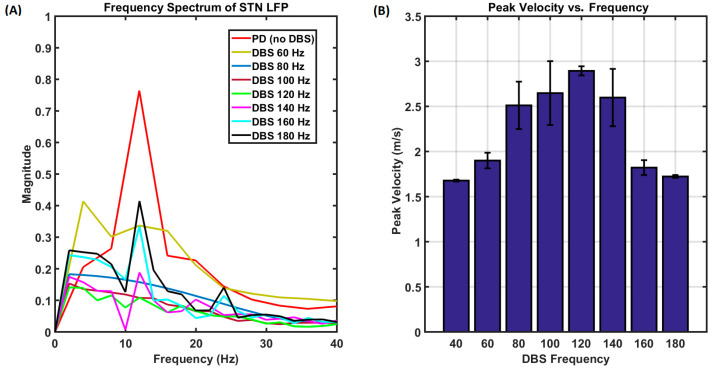
(**A**) The frequency spectra of STN LFP for PD and DBS-applied conditions are shown. (**B**) The peak velocities during a reaching task for varying DBS frequencies are shown.

High-frequency DBS stimulation of STN also seemed to alleviate the bradykinesia symptoms with a DBS pulse train of frequency 120–130 Hz with an amplitude of 200 pA, and a pulse width of 100 μS. The peak velocity of the reaching performance with the application of DBS with varying frequencies is shown in Figure 11. This is in line with the experimental results observed in [67], where the study was conducted with 10 patients with PD and showed significant improvement in bradykinesia and rigidity with a DBS stimulation frequency of 130 Hz. The recordings were taken over an average of five trials. Also, as mentioned in [67], the slowness of performance correlates with the beta peaks in our model performance. Figure 11A shows the frequency spectrum of STN LFP, and the increased beta power correlates with the reaching movements, as indicated in Figure 11B, which shows the peak velocities of the reaching movements for different DBS frequencies.

### 3.7. Comparison with Experimental Data

We also analyzed the trend in our model performance using the experimental data. As highlighted in Section 3.2, the trend in the reaching performance in the HC and PD groups were similar to that in [61]. The movement time (MT) considerably increased in patients with PD compared to that of the HCs in our model results [61]. Also, the peak velocity (PV) for PD decreased compared to that of the HC in our model results and in [61]. The time to peak velocity (TPV) also showed a similar trend in both the modeling results and the results reported in [61], with TPV being considerably higher in the case of PD. The DBS performance recorded in [63] also showed similar movement time trends and peak velocity trends for off DBS and on DBS cases, where a DBS of 130 Hz was applied for most patients. In [63], the participant was required to move the handle toward the target a few centimeters apart. For the data used for comparison in Table 1, we considered a target distance of 12 cm. The recorded MT, PV, and TPV followed the trend observed in our model. Finally, we also compared the performance of our model with the upper limb movement task in [64], and the trend was similar with movement time, reduced with high-frequency DBS on, and the PV increased with DBS on. The respective values of the model and the experimental data are given in Table 1. ‘NA’ is mentioned wherever data were unavailable.

The overall trend in Table 1 indicates that the performance deteriorates under PD conditions, which is improved with the application of DBS. Regarding dynamics, STN neurons display increased synchrony under PD conditions and exhibit higher power in the beta band. In some cases, higher power is also observed in both the beta and theta bands, especially in cases of PD tremor [70]. Our model also shows higher powers in the theta and bands in case of PD tremors, with a comparatively higher power in the theta band, which is in line with the literature data [70] (Figure 9C), and increased power in the beta band under bradykinesia and rigidity dominant conditions (Figure 11A). During the tremor condition, the beta peaks are also accompanied by another peak in the theta band, which is in line with the observations in an experimental study [68]. High-frequency DBS stimulation of the STN also seems to alleviate the symptoms of bradykinesia. This is in line with the experimental results observed in [68].

## 4. Discussion

The focus of this study was understanding the origins of PD tremors in the cortico-basal ganglia circuitry, understand the effect of DBS on PD tremors, and optimize DBS parameters to minimize tremors. As such, we developed a cortico-basal ganglia model, aiming to simulate the therapeutic effects of DBS on PD motor symptoms, most importantly PD tremors and bradykinesia. Since PD tremor was found to sensitively depend on the synchronized firing dynamics of the STN–GPe neurons [24,34,35,36,37] and as DBS action, which aims to suppress tremor, is expected to suppress the synchronized peak in the STN–GPe, in the current model, we used a spiking neuron model of the STN–GPe system.

The cortico-basal ganglia model used in this study is based on concepts from reinforcement learning and is based on some of our earlier work [22]. Central to our model is the idea that the cortico-basal ganglia system achieves movement control by performing stochastic hill-climbing over the value function. We posit this function as readily accessible within the BG, courtesy of top-down processing from the prefrontal areas, where goal-related information is available.

Leveraging this value function, we derived a value difference signal regulating the BG pathways and connections within the STN–GPe network. The value difference signal in our model acts similarly to the dopamine signal used to switch between the direct and indirect pathways via its discriminative actions on the D1 and D2 cells of the striatum. The dopamine signal also influences STN dynamics, representing the projections of the SNc to the STN. To produce Parkinsonian tremors, we manipulated this signal, mimicking reduced dopamine levels, which profoundly influence the dynamics of the STN–GPe subsystem.

Consistent with the experimental findings, our model replicates the behavioral outcomes from the experiments. The behavior was validated in terms of movement time, peak velocity, and the time to peak velocity. Also, as the experimental literature describes, our model demonstrates an increase in beta power within the STN’s local field potential (LFP) under reduced dopamine conditions [68]. The higher power is also observed in both the beta and theta bands, especially in cases of PD tremor, which is in line with the literature [37]. Our modeling results reveal that the PD tremors correlate more with the higher theta power within the STN, whereas bradykinesia correlates more with the beta power within the STN neurons. DBS stimulation of 130 Hz, 200 pA, and a 100 μS signal successfully alleviated PD tremor symptoms, whereas DBS stimulation of 120/130 Hz, 200 pA, and a 100 μS signal successfully suppressed the beta peaks in the STN LFP signal, thereby improving bradykinesia symptoms. During normal reaching movements, the velocity characteristics form a perfect bell curve, and the peak velocity is higher, while during bradykinesia, the velocity curve does not form a perfect bell curve, and the peak amplitude is also comparatively lower. Via stimulating with a DBS signal of varying frequencies, we could observe that the DBS signals with frequencies of around 130 Hz effectively suppressed the beta peaks in the STN LFP. Thus, our modeling study successfully validated the effectiveness of STN DBS for PD conditions.

However, STN DBS is not without its side effects. While DBS effectively attenuates motor symptoms, concerns persist regarding its impact on cognitive function [71,72]. Studies have reported the emergence of impulsive control disorder (ICD) following the stimulation of the STN [73,74,75,76,77,78]. The connection between STN DBS and ICD is debatable as some studies have also revealed that a small percentage of people undergoing DBS have experienced cognitive decline [79,80], whereas some studies have reported a favorable effect on cognitive performance [81,82]. Studies have reported that DBS treatment for patients with PD with neuropsychiatric disorders, including depression, anxiety, and ICD, might further worsen their cognitive performance. Hence, patients with severe pre-existing neuropsychiatric disorders should be given DBS with caution [83]. However, despite all the diverse results, it would be interesting to explore the effect of DBS treatment on motor and cognitive symptoms simultaneously.

One of the limitations of the current study is that the cortico-cerebellar pathway was not exclusively modeled. In fact, the cerebellum plays a crucial role in coordinating and executing motor movements. Refs. [47,84], in their work on learning in sequential procedures using a visuomotor task, highlight the role of both the BG and cerebellum in voluntary motor control. One of the major DBS stimulation targets for tremors, including PD tremors, is the VIM thalamus, which receives projections from the cerebellum via the dentato-rubro-thalamic tract (DRTT) [85]. This can be studied by incorporating the cerebellum into our model. While STN DBS is effective for the major cardinal symptoms of PD, such as tremors, bradykinesia, and rigidity [86], VIM DBS does not improve rigidity or bradykinesia [87]. The inclusion of cortico-cerebellar circuitry facilitates studying different DBS target areas; hence, it would be an interesting consideration to include this pathway and associated connections. Some studies have reported that PD tremor has a different origin than essential or intentional tremors. While PD tremor is attributed to an imbalance in the basal ganglia functional circuitry, essential tremor has a cerebellar origin, and, hence, it is important to look at whether PD tremor has a cerebellar origin. Pathological changes in the cerebellum could be caused by dopaminergic loss due to PD, or they could be compensatory mechanisms [88]. Taking inferences from various studies [84, 89,90,91], our understanding is that the cerebellum is primarily involved in forward prediction and error correction, processes that are relatively fast and typically engaged during routine and learned movements. In contrast, during sensory feedback perturbations, environmental changes, or novel tasks, the role of the thalamocortical-basal ganglia circuitry becomes more prominent. This is a comparatively slower process. In modeling terms, we need to incorporate faster supervised-learning-based cortico-cerebellar circuitry in parallel with slower reinforcement-learning-based cortico-basal ganglia circuitry. While proceeding with this future implementation, we may also need to explicitly model the posterior parietal cortex, which integrates sensory and proprioceptive feedback signals.

## 5. Conclusions and Future Work

Summarizing the results, in this study, we modeled cortico-basal ganglia circuitry for simulating a DBS intervention. Our model captured the kinematic performance during the reaching task and was able to match the trend observed in the experimental data. We observed that during PD, the average movement time and TPV were higher than for HCs, whereas the PV was comparatively lower in case of PD. The STN dynamics observed in the model showed higher power in the frequency spectrum of the STN LFP signal, the tremor correlated more with the theta band, and the bradykinesia correlated more with the beta band. DBS stimulation with a 130 Hz signal successfully alleviated both tremors and bradykinesia, which is in line with the literature findings. Thus, using our computational model, we successfully showed that a DBS intervention with appropriate characteristics does reduce PD symptoms. Not many experimental studies have shown distinct spectral characteristics of STN LFPs, and it would be interesting to look into these neural dynamics for different PD symptoms. As stated in the Discussion, inclusion of the cerebellum and the corticocerebellar pathway will make the model more robust, which will also facilitate exploring the computational modeling of different DBS targets. Along this line, we will be expanding our model to include this change.

**Figure 12:**
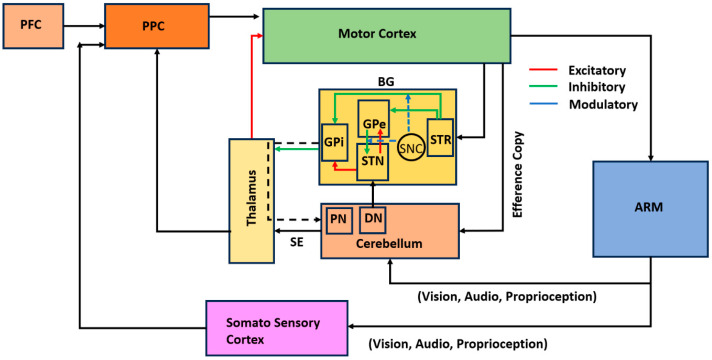
Hypothetical block diagram with the integrated cortico-basal ganglia and the corticocerebellar loops. PPC, posterior parietal cortex; STR, striatum; PN, pontine nuclei, DN, dentate nuclei; PFC, prefrontal cortex; GPe/GPi globus pallidus externus and globus pallidus interna.

The hypothetical figure for the proposed model is shown in Figure 12. The somatosensory cortex (SSC) integrates sensory and proprioceptive feedback and sends this integrated information to the posterior parietal cortex (PPC). The cerebellum computes sensory prediction errors and sends these errors to the PPC. The PPC integrates the sensory feedback from the SSC and the sensory prediction errors from the cerebellum, adjusting the action to the goal based on this integrated information. The involvement of the cortico-basal ganglia loop and the cortico-cerebellar loop in motor control varies depending on sensory prediction errors. The PPC then forwards the refined action plan to the motor cortex, which executes the movement.

Also, currently, there are not many studies that have focused on combining DBS and rehabilitation for PD. It was reported that post-DBS surgery, patients often face gait and balance issues [92]. DBS is not recommended for patients with balance problems since DBS exacerbates the gait and balance issues in patients with PD [83]. This gives an incentive to explore DBS options in combination with rehabilitation. Ref. [93] hypothesized an improvement in balance and gait if the patients undergo physiotherapy post-STN-DBS. The physical therapy exercises included trunk rotation, flexion of the hip, hip abduction, etc. Ref. [94] used robot-assisted rehabilitation for patients with PD with DBS, which showed improvements in spatiotemporal gait parameters. Ref. [95] reported improvements in the functional impairments associated with STN-DBS when the patients were subjected to rehabilitation. In the future, the DBS parameter space will receive a lot of attention when the patient undergoes DBS in combination with the rehabilitation regime.

Therefore, our next step involves refining this model to identify the nuanced interplay between DBS, motor symptoms, and cognitive function; model the DBS stimulation effects on PD symptoms in different target areas such as the STN, GPi, and VIM [20, 56,57], model multiple BG control loops; and incorporate a control loop that optimizes the amount of medication and stimulation. By meticulously regulating model parameters, we strive to simulate a wide range of patient profiles and obtain an optimized therapeutic intervention strategy at various stages of the disease. In addition to this, a combination of DBS and rehabilitation will be the next frontier. One of the key motivations behind combining DBS with rehabilitation in PD is as follows: one may expect comparable results in motor performance, even with milder DBS currents, when DBS is combined with rehabilitation.

## Figures and Tables

**Table 1:** Comparison between Model and Experimental performances.

MODEL/EXP	Category	MT (s)	PV (m/s)	TPV (s)
MODEL	HC	0.41 ± 0.048	3.18 ± 0.36	0.31 ± 0.034
	PD/DBS OFF	0.837 ± 0.084	1.49 ± 0.27	0.62 ± 0.008
	DBS ON	0.44 ± 0.028	2.89 ± 0.02	0.35 ± 0.016
EXP1 [61]	HC	0.343 ± 0.004	2.15 ± 0.27	0.19 ± 0.02
	PD/DBS OFF	0.52 ± 0.063	1.35 ± 0.18	0.27 ± 0.003
	DBS ON	NA	NA	NA
EXP2 [63]	HC	~0.8 ± 0.1	~0.33 ± 0.06	~0.446
	PD/DBS OFF	~1.35 ± 0.18	~0.175 ± 0.033	~0.756
	DBS ON	~1.05 ± 0.16	~0.25 ± 0.05	~0.637
EXP3 [64]	HC	NA	NA	NA
	PD/DBS OFF	0.505 ± 0.017	0.34 ± 0.018	NA
	DBS ON	0.46 ± 0.015	0.35 ± 0.005	NA

## Data Availability

The data presented in this study are available on request from the corresponding author due to (privacy).

## Supplementary Materials

The following supporting information can be downloaded at: https://www.mdpi.com/article/10.3390/brainsci14060620/s1.
