# Supplementary material for: A Computational Model of Deep Brain Stimulation for Parkinson’s Disease Tremor and Bradykinesia
Source: Brain Sci. 2024 Jun 20;14(6):620. doi: 10.3390/brainsci14060620 (PMC11201485; doi:10.3390/brainsci14060620)
Supplement: Supplementary file 1 [file brainsci-14-00620-s001.zip › brainsci-3011481-supplementary.pdf]

Supplementary Information

A Computational Model of Deep Brain Stimulation for Parkinson's Disease Tremor

Sandeep Sathyanandan Nair[1] , V. Srinivasa Chakravarthy[1,2 *]

* Corresponding Author:

Prof. V. Srinivasa Chakravarthy, Ph.D.

[1]Department of Biotechnology, Bhupat and Mehta Jyoti School of Biosciences,

[2]Department of Medical Science and Technology,

Indian Institute of Technology Madras, Sardar Patel Road,

Adyar, Chennai 600036, Tamil Nadu, India.

Tel: +91 44 2257 4115

Fax: +91 44 2257 4102

Email: schakra@ee.iitm.ac.in

S1. Training of the cortico-basal ganglia model

We use a cortico-BG (CBG) model that controls a two-linked arm model in order to simulate reaching movements as shown in (Figure S1). In this section we will first introduce the CBG model, the training of the outer cortico-basal ganglia loop and the model parameters. The training scheme and the model is adapted from [1], [2], [3]

1.1. Cortico-basal Ganglia Model

The CBG model used in our study comprises of an outer sensory motor loop, which interacts with the BG circuitry. The sensory motor loop comprises of an arm model, proprioceptive cortex (PC), motor cortex (MC), the prefrontal cortex (PFC) and the BG. The kinematic arm model performs reaching movements based on the activations it receives and the PC estimates the current arm position and sends the feedback to MC, which integrates this signal along with the goal information from the PFC and the error corrected signal from the BG, which does its own internal processing before sending back the corrected signal to the motor cortex. The motor cortex then sends the next motor command to the arm via the spinal motor neurons and this

process continues until the arm reaches the target or the time out is reached. More details about the sensory motor loop and the arm model are discussed in the following sections.

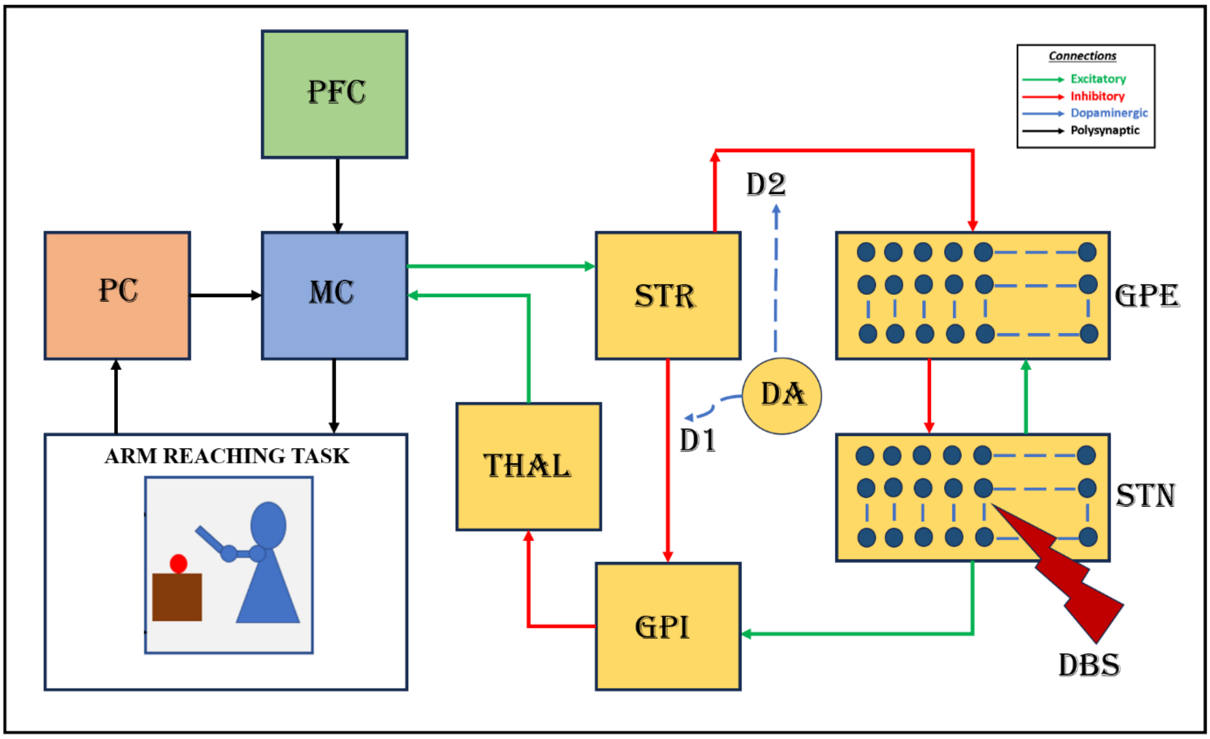

Figure S1. Block diagram of the proposed cortico-basal ganglia model. The model consists of a 2-link arm model, the proprioceptive cortex (PC), the prefrontal cortex (PFC), the motor cortex (MC), and Basal ganglia (BG). Here the input nucleus Striatum, the output nucleus Globus pallidus interna (GPi), the Globus pallidus externa (GPe), the subthalamic nucleus (STN), and the thalamus (THAL) constitute the BG. MC integrates the inputs received from the prefrontal cortex (PFC) and the proprioceptive cortex (PC) along with the feedback signal from BG and sends the signal to the arm via the spinal motor neurons.

1.1.1. Training of the outer sensory-motor loop

The training of the sensory-motor loop comprises of the below steps.

1. First, we generate n random activation vectors for the motor neurons resulting in various arm movements. This yields n different arm configurations, each providing a feature vector of muscle lengths (M^L).

2. M^L is given as input to the PC that undergoes learning using self-organizing maps to form a sensory activation map.
3. The PC output is the input to the MC, and MC output is given as input to MN so that the desired activation vector (ϕ^{MN})- initially given to the arm is obtained back thereby closing the loop.
4. In the above three steps, first the sensory activation map was formed by training the connections between the arm and PC. Then, the connections between PC and MC are trained, and in the third stage, the connections between the MC and MN are trained.
5. At the final step, BG is introduced and PFC to MC connections are trained (Figure S2).

Before discussing the details of the training mechanisms involved in the cortico-basal ganglia model used in our model, we will first define the arm model and its important parameters and then discuss how the muscle length vector is calculated.

1.1.2. Obtaining the Muscle length vector for different activations

As mentioned in the previous section the training of the model starts with obtaining the muscle length vector for various activations of the motor neurons. The arm model simulates the movement of the arm in the two-dimensional space [4], [5] (Figure S2). In our model we consider two joints – the shoulder and the elbow. These joints are controlled by an antagonist (An) and an agonist (Ag) muscle pair and the activations to these muscle groups (ϕ_{SE}^{MN}) are transformed into joint angles for both shoulder and the elbow as follows,

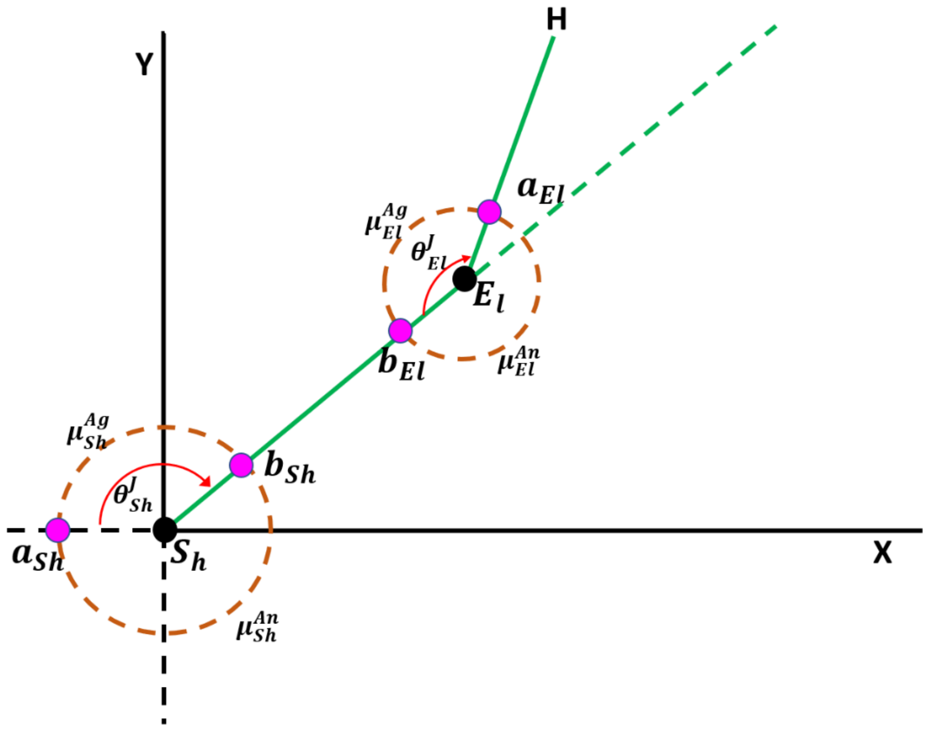

Figure S1: The rm model. *S_h and E_l are the shoulder and elbow joints; the end effector position is marked as H; The axes of the plane are labeled as X & Y; l_e and l_S are the distances between the (H and E_l) and (E_l and S_h) respectively; θ_{Sh}^{J} and θ_{El}^{J} represents the angles at the shoulder and elbow respectively; $[\mu_{Ag}^{Sh}, \mu_{An}^{Sh}, \mu_{Ag}^{El}, \mu_{An}^{El}]$ represents the muscle length vector for agonist and antagonist pairs of shoulder and elbow muscles, respectively; $[a_{Sh,} b_{Sh}, a_{El}, b_{El}]$ represents the distance between (S_h and M_1 or M_2 moment lever) and (S_h and M_3 or M_4 moment lever) respectively.*

$$\theta_{Sh}^{J}(t) = \left(\phi_{Sh_{Ag}}^{MN}(t) - \phi_{Sh_{An}}^{MN}(t)\right)\frac{\pi}{2} + \frac{\pi}{2} \tag{1}$$

$$\theta_{El}^{J}(t) = \left(\phi_{El_{Ag}}^{MN}(t) - \phi_{El_{An}}^{MN}(t)\right)\frac{\pi}{2} + \frac{\pi}{2} \tag{2}$$

where, θ_{El}^{J} and θ_{Sh}^{J} are the elbow and shoulder joint angles with respect to the horizontal axis (Figure S1) and two-dimensional shoulder length (l_{Sh}) respectively. $\phi_{S_{Ag}}^{MN}$, $\phi_{S_{An}}^{MN}$, $\phi_{El_{Ag}}^{MN}$ and $\phi_{El_{An}}^{MN}$ are the muscle activation of the agonist and the antagonist muscles of the shoulder and elbow muscles respectively.

The joint angles define the coverage of the arm in the two-dimensional space and are used to calculate the muscle lengths as given in equations (3-6) below. This process is represented in (Figure S3)

$$\mu_{Ag}^{Sh}(t) = \sqrt{a_{Sh}^2 + b_{Sh}^2 + 2a_{Sh}b_{Sh}\cos(\theta_S^J)} \quad (3)$$

$$\mu_{An}^{Sh}(t) = \sqrt{a_{Sh}^2 + b_{Sh}^2 - 2a_{Sh}b_{Sh}\cos(\theta_{Sh}^J)} \quad (4)$$

$$\mu_{Ag}^{El}(t) = \sqrt{a_{El}^2 + b_{El}^2 + 2a_{El}b_{El}\cos(\theta_{El}^J)} \quad (5)$$

$$\mu_{AN}^{El}(t) = \sqrt{a_{El}^2 + b_{El}^2 - 2a_{El}b_{El}\cos(\theta_{El}^J)} \quad (6)$$

where, μ_{Ag}^{Sh}, μ_{An}^{Sh}, μ_{Ag}^{El}, and μ_{An}^{El} are the agonist and antagonist muscle lengths of shoulder and elbow, respectively, a_{Sh} and b_{Sh} are the distance between shoulder joint center and M_1 or M_2 moment lever, and, a_{El} and b_{El} are the distance between elbow joint center and M_3 or M_4 moment lever respectively.

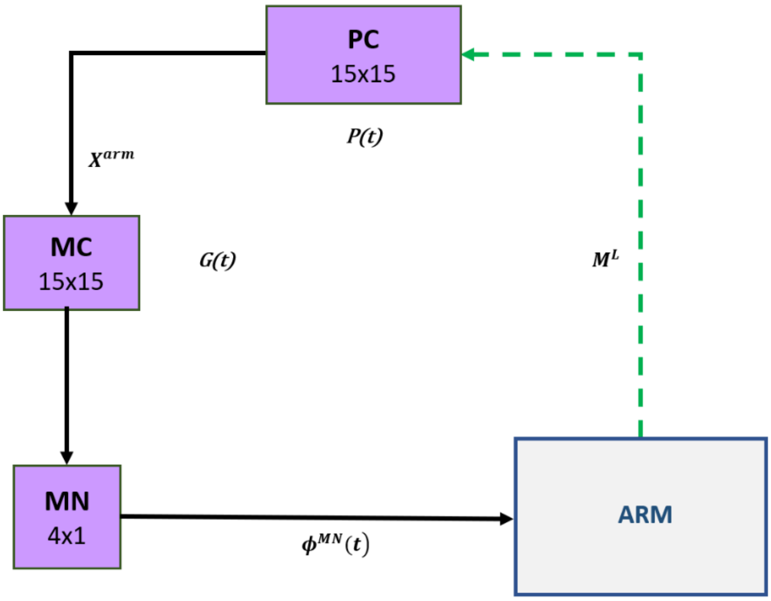

Figure S3: The training schema for ARM-PC connections (Sensory-motor loop). *In the figure, the dashed arrows indicate the connections that are being trained. PC, proprioceptive cortex; MC, motor cortex; MN, motor neuron; PFC, prefrontal cortex; BG, basal ganglia;* X^{arm}*, the current arm position;* ϕ^{MN}*, the motor neuron activations;* M^L*, muscle lengths;* $P(t)$*, the PC output;* $\Delta G(t)$*, the MC output.*

Using these muscle lengths in the form of a four-dimensional vector $(M^L = [\mu_{Ag}^{Sh}\ \mu_{An}^{Sh}\ \mu_{Ag}^{El}\ \mu_{An}^{El}])$, a sensory (proprioceptive) map of the arm was generated. The end effector position of the arm $(X^{arm} = [x_1^{arm}\ x_2^{arm}])$ in the two-dimensional space is calculated as shown in equations (7-8),

$$x_1^{arm} = (l_{Sh} - a_{Sh})\cos(\theta_{Sh}^J) + l_{El}\cos(\theta_{Sh}^J + \theta_{El}^J) \tag{7}$$

$$x_2^{arm} = (l_{Sh} - a_{Sh})\sin(\theta_{Sh}^J) + l_{El}\sin(\theta_{Sh}^J + \theta_{El}^J) \tag{8}$$

$$M^L = [\mu_{Ag}^{Sh}\ \mu_{An}^{Sh}\ \mu_{Ag}^{El}\ \mu_{An}^{El}] \tag{9}$$

where, θ_{Sh}^J and θ_{El}^J are the joint angles of shoulder and elbow with respect to the horizontal axis (Figure S1) and shoulder length (l_{Sh}), respectively in two-dimensional space, l_{Sh} is the distance between the shoulder joint center (S) and elbow joint center (E), l_{El} is the distance between the elbow joint center (E) and end effector (H). M^L is the muscle length vector.

In order to train the PC to MC connections as shown in (Figure S4), we need to first obtain the sensory activation map and the activation map for the PFC. These two processes are discussed in subsections (1.1.3 and 1.1.4) below.

1.1.3. Obtaining the sensory activation maps

Using the M^L computed above, we proceed with obtaining the sensory activation map. PC receives the input from the arm model and it sends the feedback signal to the MC. It is modelled as a self-organizing map (SOM) [6] where sensory map of the arm is created. Below equation (10) shows the activity of the single neuron of the PC,

$$U_{ij}^{PC}(t) = exp\left(\frac{-\left\|M^L(t) - W_{ij}^{PC}\right\|^2}{(\sigma^{PC}).(\sigma^{PC})}\right) \tag{10}$$

where, W_{ij}^{PC} is the weight between M^L and $(i,j)^{th}$ neuron of the two-dimensional PC network, M^L is the muscle length vector and the spatial distance within which the response of the PC SOM shows sensitivity is given by σ^{PC}.

1.1.4. Training of the Prefrontal Cortex (PFC)

PFC provides the goal position. Biologically the goal position is obtained by the visual feedback, where as in our model we fix the goal position as X^{targ}. The initial signal from the motor cortex (MC) to motor neurons (MN) is initially facilitated by the PFC and gradually with learning the BG circuitry takes over. All possible locations where the arm has the possibility

to reach constitutes the features of PFC. The PFC is trained using the target vector and the activity of neuron at $(i, j)^{th}$ location in the PFC is given as in equations (11).

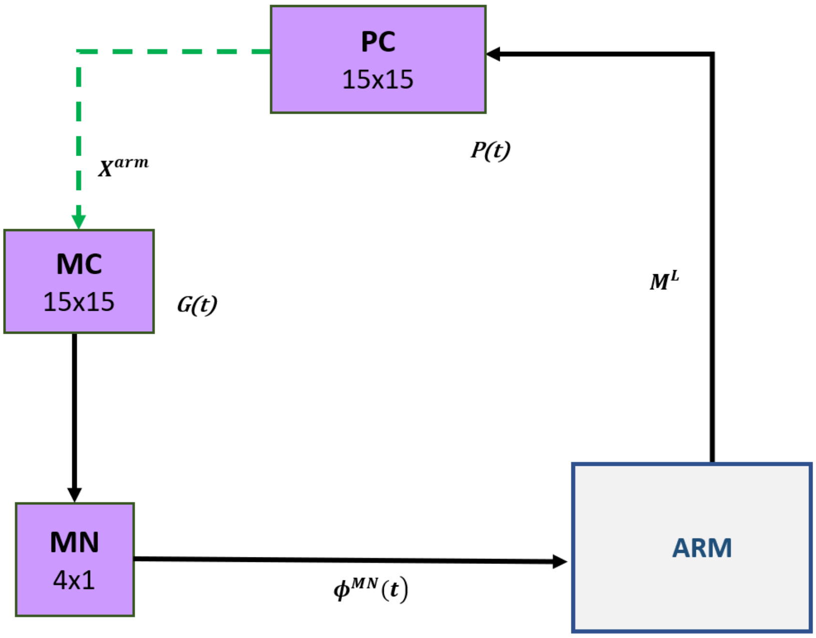

Figure S3: The training schema for PC-MC connections (Sensory-motor loop). *In the figure, the dashed arrows indicate the connections that are being trained. PC, proprioceptive cortex; MC, motor cortex; MN, motor neuron; PFC, prefrontal cortex; BG, basal ganglia;* X^{arm}*, the current arm position;* ϕ^{MN}*, the motor neuron activations;* M_L*, muscle lengths;* $P(t)$*, the PC output;* $\Delta G(t)$*, the MC output.*

$$U_{ij}^{PFC}(t) = exp\left(\frac{-\left\|X^{targ}(t) - W_{ij}^{PFC}\right\|^2}{(\sigma^{PFC}).(\sigma^{PFC})}\right) \tag{11}$$

W_{ij}^{PFC} is the weight between the target position vector and $(i, j)^{th}$ neuron and the 2-D PFC network and σ^{PFC} is the width of the PFC SOM response.

1.1.5. Training MC to Motor neuron (MN) connection

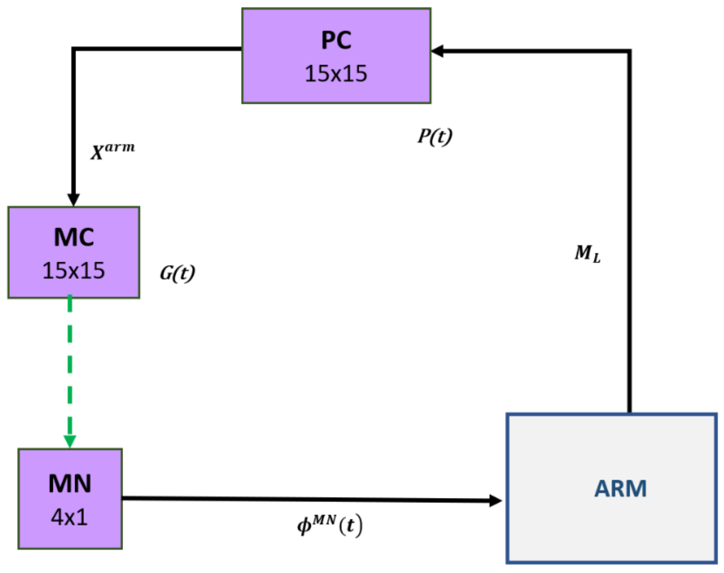

Figure S5: The training schema for MC-MN connections (Sensory-motor loop). *In the figure, the dashed arrows indicate the connections that are being trained. PC, proprioceptive cortex; MC, motor cortex; MN, motor neuron; PFC, prefrontal cortex; BG, basal ganglia;* X^{arm}*, the current arm position;* ϕ^{MN}*, the motor neuron activations;* M_L*, muscle lengths;* $P(t)$*, the PC output;* $\Delta G(t)$*, the MC output.*

The MC output projects to the four MN neurons, which drives the muscle activations in order to make the arm movements. The activation of the MN layer is given by equation (12),

$$\phi^{MN} = A^{MN} W^{MC \to MN} G^{MC}(t) \tag{12}$$

where, A^{MN} is the gain of MN, $W^{MC \to MN}$ is the weight matrix between MC and MN layer, and G^{MC} is the output activity of MC CANN.

To close the sensory-motor loop, we perform a comparison with the initial activation to the MN layer that was used to produce desired activation $\phi_D^{MN}(t)$. The weights between the MN and MC are trained in a supervised manner by comparing the network derived MN activation $\phi^{MN}(t)$ to the desired activation $\phi_D^{MN}(t)$. This gives a loop which is consistent in mapping the external arm space to the neuronal space and vice. The connection between MC and MN is trained as represented in (Figure S5) and is given by equation (13),

$$\Delta W^{MC \to MN} = \eta^{MC \to MN} \left(\phi_D^{MN}(t) - \phi^{MN}(t) \right) G^{MC}(t) \tag{13}$$

where, $\eta^{MC \to MN}$ is the learning rate between MC and MN, ϕ_D^{MN} is the desired MN activation required for the arm to reach the target and ϕ^{MN} is the network-derived MN activation due to

MC, and G_{MC} is the output activity of MC. The training schema for the outer loop (sensory-motor loop) is described in section S3 of the supplementary information.

1.1.6. Motor Cortex (MC) and MC to PFC training

The MC receives inputs from the PFC, the PC and the BG. MC is modeled as a combination of self-organizing map and continuous attractor neural network (CANN) [7]. The total input received at the MC, I^{MC}, is contributed by the PC, the PFC and the BG as shown in equation (14) below.

$$I^{MC} = A^{PFC}.G^{PFC} + A^{PC}.G^{PC} + A^{BG}.G^{BG} \tag{14}$$

where, (A^{PFC}, A^{PC}, A^{BG}) and (G^{PFC}, G^{PC}, G^{BG}) represents the gains and output activities of PFC, PC and BG respectively. The PC activity used to generate feature maps in MC is given below where the activation of the $(i,j)^{th}$node in the SOM part of the MC $\left(G_{PC,ij}\right)$ is given as in equation (15) below,

$$G_{PC,ij}(t) = exp\left(\frac{-\left\|U^{PC}(t) - W_{ij}^{MC}\right\|^2}{(\sigma^{MC}).(\sigma^{MC})}\right) \tag{15}$$

where, U^{PC} is the output activity of PC SOM network, W_{ij}^{MC} is the weight between the PC network and $(i,j)^{th}$ neuron of the two-dimensional MC network, and the spatial distance within which the response of the MC SOM shows sensitivity is given by σ^{MC}.

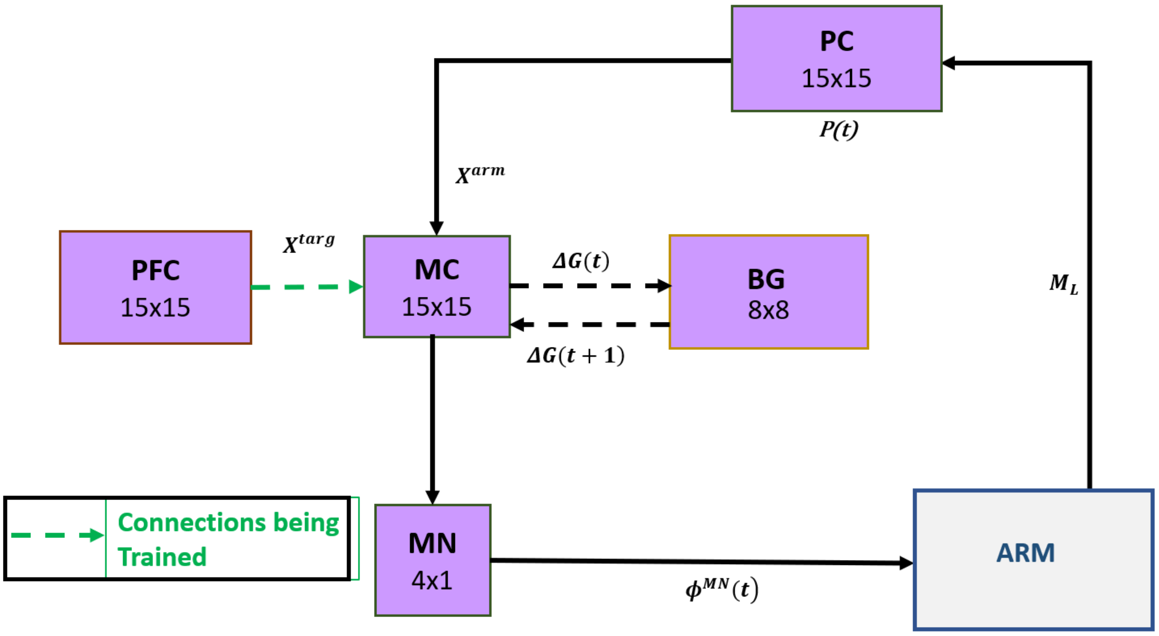

Figure S6: The training schema for PFC to MC connections (sensory-motor loop. *BG module is introduced and the PFC to MC connections are trained. The dashed arrows indicate the connections that are being trained. PC, proprioceptive cortex; MC, motor cortex; MN, motor neuron; PFC, prefrontal cortex; BG, basal ganglia;* X^{targ}*, the target position;* X^{arm}*, the current arm position;* ϕ^{MN}*, the motor neuron activations;* M_L*, muscle lengths;* $P(t)$*, the PC output;* $\Delta G(t)$*, the MC output;* $\Delta G(t+1)$*, the BG-derived activity of thalamus.*

The input from PFC to MC (G_{PFC}) is the product of weight matrix ($W^{PFC\rightarrow MC}$) and the output activity of PFC SOM is given by equation (16) below,

$$G_{PFC}(t) = W^{PFC\rightarrow MC} * U^{PFC}(t) \tag{16}$$

where, U^{PFC} is the output activity of PFC SOM network, $W^{PFC\rightarrow MC}$ is the weight matrix between PFC and MC.

The output of MC is defined by equation (17) below,

$$G^{MC}(t) = \frac{\left(g^{MC}\right)^2}{1+\left(\frac{2\pi}{\left(N^{MC}\right)^2}\right) b^{MC} \sum \left(g^{MC}\right)^2} \tag{17}$$

where, N^{MC} defines the size of the network, b^{MC} is a constant and g^{MC} represents the intrinsic dynamics of the MC neurons and it is as given in the equation (18) below,

$$\tau_{MC} \frac{dg^{MC}}{dt} = -g^{MC} + W_{MC}^{C} \otimes G^{MC} + I^{MC} \tag{18}$$

where, the weight kernel W_{MC}^{C} represents the lateral connectivity among the MC neurons as given in equation (19), whose dynamics are defined by local excitation and global inhibition, and ⊗ represents the convolutional operation.

$$W_{MC,i,j}^{C} = A_{lat}^{C} \exp\left(\frac{-\|(i^{MC} - i^{h}) + (j^{MC} - j^{h})\|^2}{2(\sigma_{lat}^{C})^2}\right) - K^{C} \tag{19}$$

where, $[i^{MC}, j^{MC}]$ are the position of the neurons in MC, $\left[i^{h}, j^{h}\right]$ is the position of the neuron in the centre, A_{lat}^{C} is the strength of the excitatory connections, K^{C} is the global inhibition constant and σ_{lat}^{C} is the radius of the excitatory connections.

When the arm reaches the target ($\epsilon < 0.1$), the connections between the PFC and MC are trained and is as given in equation (20) below,

$$\Delta W_{PFC\rightarrow MC} = \eta_{PFC\rightarrow MC} \left(G_{targ}^{MC}(t) - G_{PFC}^{MC}(t)\right) U^{PFC}(t) \tag{20}$$

where, $\eta_{PFC\rightarrow MC}$ is the learning rate between PFC and MC, G_{targ}^{MC} is the MC activation required for the arm to reach the target, and $G_{PFC}^{MC}(G_{PFC})$ is the MC activation due to PFC.

2 The Model parameters

The model parameters used in our model is as given in Table S1 below. The parameters [a^{gpe}, b^{gpe}, c^{gpe}, d^{gpe}] and [a^{STN}, b^{STN}, c^{STN}, d^{STN}] represents the Izhikevich parameters using which the spiking neurons were modeled. I_{STN} and I_{GPe} are the STN and GPe currents. $E_{AMPA/NMDA/GABA}$ are the resting membrane potentials of different receptors associated with STN and GPe. $\tau_{AMPA/NMDA/GABA}$ are the time decay constants of the receptors. W_{STN}^{max} and W_{GPe}^{max} are the maximum collateral synaptic strength. DA is the dopamine signal, $A^{D1} and\ A^{D2}$ are the synaptic weight between D1 striatum and GPi and synaptic weight between D2 Striatum and GPe respectively.

Table 1. Model parameters

Parameter	Values with description

	STN		GPe
Izhikevich parameters	a^{gpe}=0.005, b^{gpe}=0.265, c^{gpe}=-65, d^{gpe}=1.5		a^{STN}=0.1, b^{STN}=0.2, c^{STN}=-65, d^{STN}=2
External current (I)	I_{STN} = 20 pA		I_{GPe} = 4.5 pA
A^{D1}	7.5		Synaptic weight between D1 striatum and GPi
A^{D2}	2 (HC and Tremor); (<0.4) Rigidity		Synaptic weight between D2 striatum and GPe
A^{GPe}	1		
DA	0.1 to 0.9 in increments of 0.1		
Mg^{2+}	1nM	Concentration of Magnesium ions in nM	
E_{AMPA}	0mv	Synaptic potential of AMPA receptor-associated channel	
E_{NMDA}	0mV	Synaptic potential of NMDA receptor-associated channel	
E_{GABA}	-60mV	Synaptic potential of GABA receptor-associated channel	
τ_{AMPA}	6ms	Time decay constant for AMPA receptor	
τ_{NMDA}	160ms	Time decay constant for NMDA receptor	
τ_{GABA}	4ms	Time decay constant for GABA receptor	
W_{GPe}^{max}	1	Synaptic strength within GPe laterals	
W_{STN}^{max}	0.51	Synaptic strength within STN laterals	

Table 2. Parameters – Behavioral Manifestation

Behavior Manifestation					
Parameter	Parameter Description	HC	Bradykinesia	Rigidity	Tremor
DA_{low}	0.5	No limit	0.5	0.5	0.5
W_{STN}^{max}	Peak lateral strength (STN)	1	0.11	0.11	2
W_{GPe}^{max}	Peak lateral strength (GPe)	1.5	1	1	3
R_{STN}	Radius of the gaussian defining the lateral connectivity among STN neurons	0.11	10	10	2
R_{GPe}	Radius of the gaussian defining the lateral	20	0.22	0.22	10

	connectivity among STN neurons				
W_{SG}	Strength of interconnectivity weights between STN and GPe	9	7.92	7.92	10
W_{GS}	Strength of interconnectivity weights between GPe and STN	210	29.7	5	1

Table 3. Parameters – STN Dynamics

**STN Dynamics**				
Parameter	Parameter Description	Normal	Theta + Beta band power spectrum	Single Beta band power spectrum
σ_s	Peak amplitude among lateral STN connections	1	2	0.11
σ_g	Peak amplitude among lateral GPe connections	1.5	3	1
R_{STN}	Radius of the gaussian defining the lateral connectivity among STN neurons	0.11	2	5
R_{GPe}	Radius of the gaussian defining the lateral connectivity among STN neurons	20	10	0.22
W_{SG}	Strength of interconnectivity weights between STN and GPe	9	10	7.92
W_{GS}	Strength of interconnectivity weights between GPe and STN	210	1	29.7

Table 4. Parameters – DBS parameters

DBS parameters			
Parameter	Parameter Description	Tremor	Bradykinesia/Rigidity
A	Amplitude	200 pA	200 pA
f	Frequency	130 Hz	100-130 Hz
PW	Pulse Width	100 uS	100 uS
$Location$	Target Location	STN	STN

S2. Synchrony

The synchronization measure, R^{syn}, is calculated using the instantaneous phases of the neurons in the subpopulation of STN and GPe neurons. R^{syn} gives a measure of the correlation among the neurons. In healthy state (HC), the synchrony will be less, while in the disease state the synchrony will be high. Park et al (2011) report the presence of intermittent synchrony between STN neurons and its Local field potentials (LFP), recorded using multiunit activity electrodes from PD patients undergoing DBS surgery (Park, Worth et al. 2011). They also calculated the duration of synchronized and desynchronized events in neuronal activity by estimating transition rates, which were obtained with the help of first return maps plotted using phase of neurons [8], [9] . To observe how dopamine changes synchrony in STN-GPe, we calculated the phases of individual neurons as defined in [10] .

The phase of jth neuron was calculated as follows,

$$\phi_j(t) = 2.\pi.\frac{(T_{j,k} - t_{j,k})}{(t_{j,k+1} - t_{j,k})} \quad (21)$$

$$R^{syn}(t).e^{i\theta(t)} = (1/N)\sum_{j=1}^{N} e^{i\phi_j(t)}) \quad (22)$$

where, $t_{j,k}$ and $t_{j,k+1}$ are the onset times of kth and $k+1^{th}$ spike of the jth neuron $T_{j,k} = [t_{j,k}, t_{j,k+1}]$, $\phi_j(t)$= Phase of jth neuron at time 't', R^{sync} is the synchronization measure $0 \leq R^{sync} \leq 1$, θ=Average phase of neurons, N=total number of neurons in the network.

$t_{j,k}$

[1] V. S. Chakravarthy and A. A. Moustafa, *Computational Neuroscience Models of the Basal Ganglia*, vol. 15, no. 5. in Cognitive Science and Technology, vol. 15. Singapore: Springer Singapore, 2018. doi: 10.1007/978-981-10-8494-2.

[2] S. S. Nair, V. R. Muddapu, and V. S. Chakravarthy, "A Multiscale, Systems-Level, Neuropharmacological Model of Cortico-Basal Ganglia System for Arm Reaching Under Normal, Parkinsonian, and Levodopa Medication Conditions," *Front Comput Neurosci*, vol. 15, 2022, doi: 10.3389/fncom.2021.756881.

[3] V. Muralidharan, A. Mandali, P. P. Balasubramani, H. Mehta, V. Srinivasa Chakravarthy, and M. Jahanshahi, "A Cortico-Basal Ganglia Model to Understand the Neural Dynamics of

Targeted Reaching in Normal and Parkinson's Conditions BT - Computational Neuroscience Models of the Basal Ganglia," V. S. Chakravarthy and A. A. Moustafa, Eds., Singapore: Springer Singapore, 2018, pp. 167–195. doi: 10.1007/978-981-10-8494-2_10.

[4] J. Izawa, T. Kondo, and K. Ito, "Biological arm motion through reinforcement learning," *Biol Cybern*, vol. 91, no. 1, pp. 10–22, Jul. 2004, doi: 10.1007/s00422-004-0485-3.

[5] M. Zadravec and Z. Matjačić, "Planar arm movement trajectory formation: An optimization based simulation study," *Biocybern Biomed Eng*, vol. 33, no. 2, pp. 106–117, Jan. 2013, doi: 10.1016/j.bbe.2013.03.006.

[6] T. Kohonen, *Self-Organizing Maps*, 3rd ed., vol. 30. in Springer Series in Information Sciences, vol. 30. Berlin, Heidelberg: Springer-Verlag Berlin Heidelberg, 2001. doi: 10.1007/978-3-642-56927-2.

[7] T. P. Trappenberg, "Continuous Attractor Neural Networks," in *Recent Developments in Biologically Inspired Computing*, IGI Global, 2011, pp. 398–425. doi: 10.4018/978-1-59140-312-8.ch016.

[8] C. Park, R. M. Worth, and L. L. Rubchinsky, "Fine temporal structure of beta oscillations synchronization in subthalamic nucleus in Parkinson's disease," *J Neurophysiol*, vol. 103, no. 5, 2010, doi: 10.1152/jn.00724.2009.

[9] C. Park, R. M. Worth, and L. L. Rubchinsky, "Neural dynamics in Parkinsonian brain: The boundary between synchronized and nonsynchronized dynamics," *Phys Rev E Stat Nonlin Soft Matter Phys*, vol. 83, no. 4, 2011, doi: 10.1103/PhysRevE.83.042901.

[10] P. F. Pinsky and J. Rinzel, "Synchrony measures for biological neural networks," *Biol Cybern*, vol. 73, no. 2, 1995, doi: 10.1007/BF00204051.
